# Surgical Anatomy of Large Retroperitoneal Teratomas in Infants: Report of Two Cases

**DOI:** 10.4137/ccrep.s907

**Published:** 2008-07-31

**Authors:** Ralf-Bodo Tröbs, Christian Geyer, Wolfgang Hirsch, Andrea Tannapfel

**Affiliations:** 1 Department of Pediatric Surgery, Marienhospital Herne, Ruhr-University of Bochum, Widumer Str. 8, D-44627 Herne, Germany.; 2 Department of Pediatric Surgery, University of Leipzig Medical Center, Liebigstr. 20, D-04103 Leipzig, Germany.; 3 Division of Pediatric Radiology, University of Leipzig Medical Center, Liebigstr. 20, D-04103 Leipzig, Germany.; 4 Institute of Pathology, BG Clinic Bergmannsheil, Ruhr-University of Bochum, Bürkle-de-la-Camp-Platz 1, D-44789 Bochum, Germany.

**Keywords:** teratoma, retroperitoneal, retropancreatic, germ cells, anatomy

## Abstract

We report on two infants of 11 and 12 months of age, respectively, with large solid-cystic retroperitoneal tumors. Complete resection was achieved, and both children are doing well over a follow-up of more than 17 months. The presented paper focuses on surgical anatomy of this very rare type of tumor. Teratomas were located mainly within the upper abdominal cavity, and both tumors displaced the pancreas in an anterior position. The following anatomical features were observed (1) displacement of surrounding organs, (2) deformation and elongation of large retroperitoneal vessels, (3) fibrous incorporation of large vessels by the tumor pseudocapsule, (4) wrapping of anterior aortic branches by lobes and fingers of the tumor.

## Introduction

Teratoma is an embryonic neoplasm derived from totipotential cells that contain tissue from at least two or more often three germ layers in variable proportions (Rowe et al. 1995; Gatcombe et al. 2004; Laberge et al. 2005; Harms et al. 2006). It may contain mature and immature tissue components as well as malignant elements. Retroperitoneal teratoma is very rare, and it occurs in only 3%–5% of teratomas at all. Despite the availability of advanced imaging techniques the pediatric surgeon should have in mind the expected anatomy of this type of tumor (Brasch et al. 1978). In a pioneer study by Hansmann and Budd (1932) retroperitoneal teratomas together with other types of tumors were described as “unattached retroperitoneal tumors” (Hansmann and Budd, 1932; Luo et al. 2005). The aim of our study was to detailed information about the growth pattern and surgical anatomy of this type of neoplasm. Furthermore, anatomical relationship to the pancreas was pointed out.

## Case Reports

### Case one

**Figure 1:**
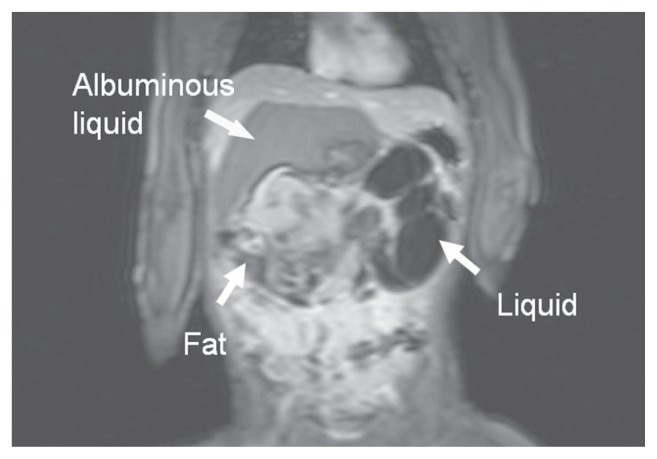
Solid—cystic teratoma containing different types of tissue like fat, albuminous liquid and other solid parts (MRI, T1-gradient echo sequence, coronal).

**Figure 2:**
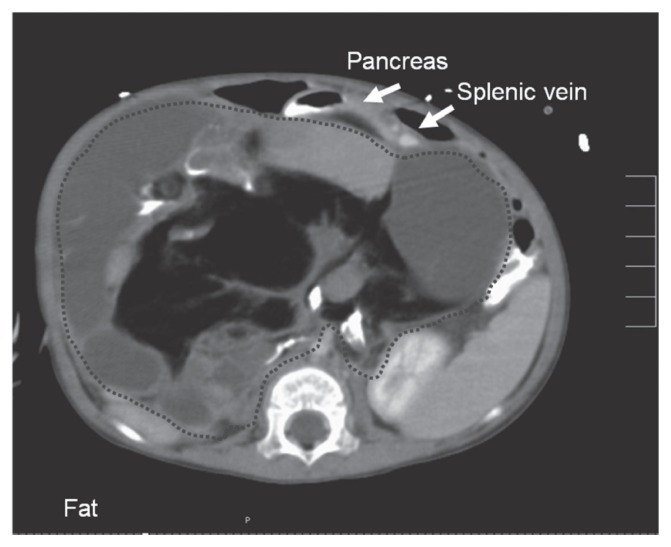
The same tumor as in Figure 1 in a transversal section. Pancreas and splenic vein are displaced towards ventral. (MRI, T1, transversal).

The twelve-month-old girl was referred to our hospital with a large abdominal protrusion. There was neither any appetite loss nor vomiting. On physical examination a mass of 20 cm in diameter was palpable within the whole upper abdomen. Serum AFP level (22 ng/ml) was at the upper border of the age related confidence interval. Ultrasound confirmed the presence of a large heterogeneous abdominal mass. Magnet Resonance Imaging (MRI) revealed a fat containing, solid and cystic retroperitoneal tumor with broad contact to the large vessels (Fig. 1). Furthermore, spleen and pancreas were displaced in an anterior position (Fig. 2). Chest CT excluded any pulmonary filiations.

**Figure 3:**
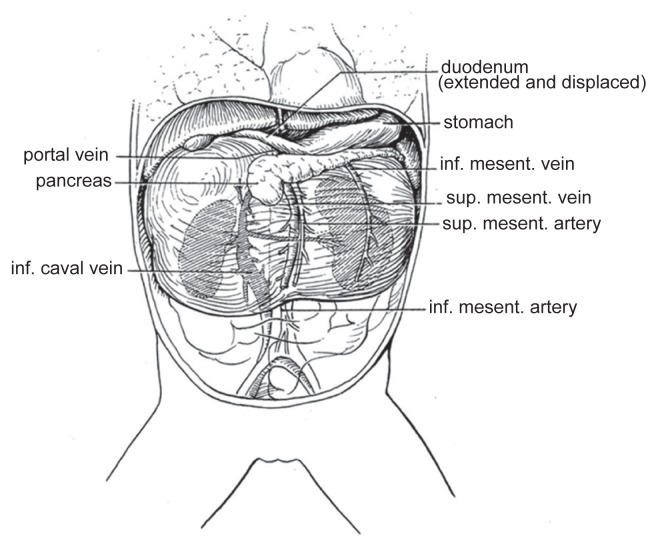
Anatomy of case one.* *Many thanks to Doctor K. Welt for his drawing.

At surgery the bulky tumor occupied the whole retroperitoneal space of the upper and middle abdomen. The maximum of the tumor was found in the right suprarenal region and protruded through the foramen of Winslow. The pancreas was flattened and stretched over the cranial portion of the tumor. Tab. 1 outlines the anatomical relationships and landmarks. The tumor was resected totally. A schematic drawing of the topographic anatomy is given in Figure 3. *Pathological findings*: The cut surface the was characterized by yellowish parenchyma, skin-like tissue, and a mature bone with marrow. Microscopic investigation of the 15 × 10 × 10 cm measuring tumor revealed the presence of mature derivates from all three germ layers, for example fatty tissue, epidermis, peripheral nerve, cartilage, teeths, intestinal mucosa, and intestine (Grade 0). The TNM tumor stage was T2b N0 M0.

After surgery the girl recovered within a couple of days. Repeated controls of serum AFP stayed within normal limits. The girl had sufficient gain of weight. Repeated ultrasound controls and estimations of serum AFP excluded tumor relapse. The girl is doing well and free of disease for 16 months postoperatively.

### Case two

**Figure 4:**
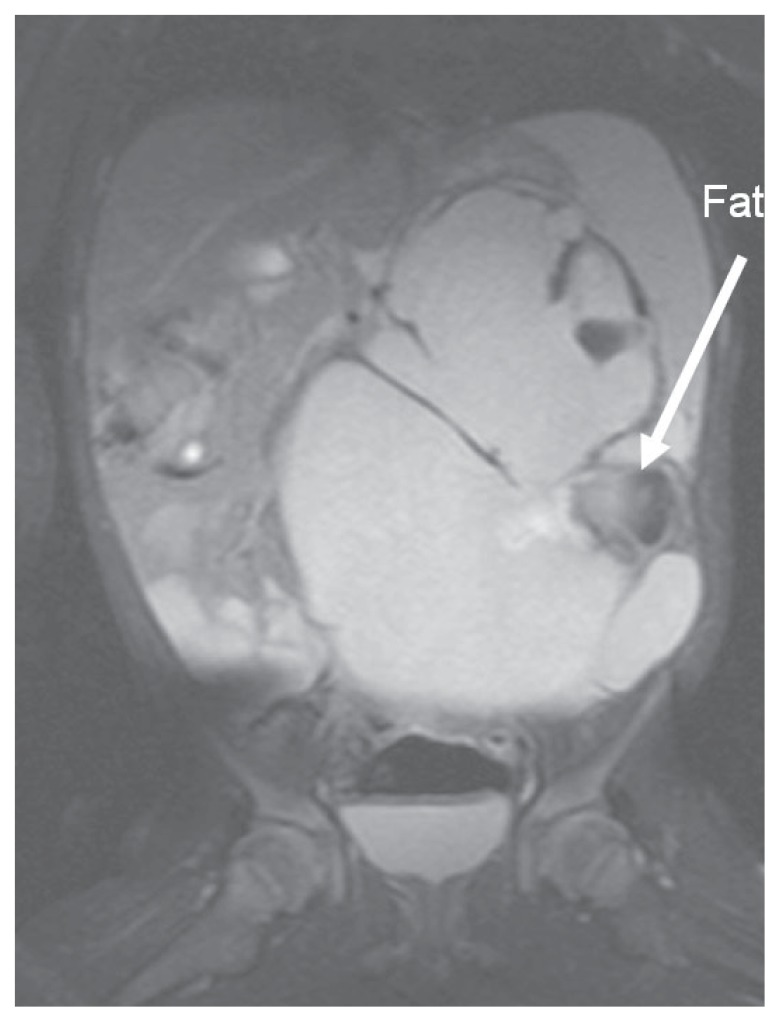
Zystic-solid teratoma in the left abdomen (case 2; MRI, T1, coronal).

The eleven-month-old Vietnamese boy was admitted in our department with a large bulky tumor of the abdomen. Previous clinical history was unremarkable. AFP was normal (15 ng/ml). The boy had bilaterally descended testes. Ultrasonography showed a large cystic-solid tumor located within the upper abdomen (Fig. 4). Abdominal MRI confirmed a large mass with fatty components and broad contact to the large retroperitoneal vessels behind the mass. The mass displaced the left kidney in a more lateral position, whereas the upper part of the tumor extended to the retropancreatic space and elevated pancreas and stomach. The top of the mass protruded caudally to the transverse colon, and it occupied nearly the whole upper and middle floor of abdominal cavity. The tumor was completely excised en bloc. *Pathological findings*: The excised tumor measured 12 × 11 × 6 cm and the cut surface mainly showed a cystic pattern. Microscopic investigation revealed the presence of mature derivates of all three germ layers (grade0). The TNM tumor stage was T2b N0 M0.

Postoperatively the boy recovered quickly. Repeated ultrasound investigations and serum AFP measurements revealed normal results. The boy is doing well over a follow-up of 17 months.

## Discussion

Retroperitoneal teratoma is the third most common retroperitoneal tumor in the pediatric population after neuroblastoma and Wilms’ tumor (Gatcombe et al. 2004). Irrespective of the presence of immature or malignant components complete surgical resection is curative in the majority of cases (Tapper and Lack, 1983; Lack et al. 1985; Billmire and Grosfeld, 1986; Marina et al. 1999; Costero-Barrios and Lopez-Briano, 2003; Gatcombe et al. 2004; Luo et al. 2005). Previous studies have reported variably an anatomic predilection for the left side, for the midline, and for a suprarenal location (Gatcombe et al. 2004). In our cases teratomas seemed to originate from the left, respectively right suprarenal region. As a consequence, the pancreas was found in front of the teratoma of both cases. Retropancreatic extension is a well known feature of adrenal neuroblastoma and left sided nephroblastoma (Kiely, 2005). In concern with teratoma it has been described occasionally in previous papers (Lack et al. 1985). For differential diagnosis mature teratoma of pancreas and the adrenal gland has been reported (Khong et al. 2002; Yu et al. 2003). However, there were clear surgical plains between the pancreas and the tumor. Furthermore, adrenal tissue was not found in any of both tumors described in this paper.

The retropancreatic extension of teratoma can be explained by the germ cell theory. During early embryonic development totipotential germ cells migrate from their normal origin in the yolk sac along the posterior mesentery of the hindgut to the gonadal ridge and then to the gonad. Incomplete or erroneous embryonic germ cell migration might occur and can be the origin of the teratoma (Gatcombe et al. 2004; Laberge et al. 2005). This process is accompanied by a clockwise rotation of the foregut including the common bile duct and ventral pancreas and resulting in fusion of the ventral and dorsal pancreatic primordia in front of the germ cells (Collins, 2002). Imaging studies of mature retroperitoneal teratomas have reported heterogeneity of the mass. Solid-cystic teratomas are complex neoplasms containing well circumscribed fluid components, fatty regions (adipose tissue and sebum in the form of a fat-fluid-level), and calcifications (Figs. 1 and 2) (Gatcombe et al. 2004). Mature teratomas are described as well circumscribed masses, showing no evidence of invasion, and seem to affect adjacent organs simply by virtue of their bulk (Davidson et al. 1989). In addition, retropancreatic localization of the teratoma, as it was found by imaging and confirmed during operation in our two cases, seems to be a characteristic feature.

The main step of therapy for retroperitoneal teratoma is complete surgical resection and the prognosis for children with retroperitoneal teratoma depends primarily upon the adequacy of surgical resection (Tapper and Lack, 1983; Billmire and Grosfeld 1986; Marina et al. 1999; Gatcombe et al. 2004; Laberge et al. 2005; Luo et al. 2005). In addition, successful laparoscopic removal was reported (Gatcombe et al. 2004). Tapper and Lack (1983) described an advanced histological grade correlates with larger tumor size and, hence, unresectability (Tapper and Lack, 1983). Incomplete resection predisposes to tumor relapse. Furthermore, the presence of small areas of malignant cells, most commonly microfoci of yolk sac tumor within the teratoma may result in a high risk of recurrence (Gobel et al. 1997; Laberge et al. 2005; Harms et al. 2006). Recurrence and late malignant transformation of retroperitoneal teratomas have been observed and require careful long-term follow-up with ultrasonography and serum AFP measurements for all patients.

In order to prevent tumor spillage and to accomplish complete removal adequate exposure of the tumor is essential. Surgery may be time consuming and requires exact proceeding between tissue planes. Table 1 summarizes the complex anatomical relationships of large retro-peritoneal teratomas. The aim of surgery is to remove all tumor tissue. As well known from other retroperitoneal tumors identification and clearing of large vessels are the most important steps of surgery (Kiely, 2005). The surgeon should keep in mind that the large aortic branches as well as the venous roots of cava vein and the portal vein have to be identified and preserved. Furthermore, different rare anomalies and malformations of the inferior vena and its branches may occur and if unrecognized can lead to life-threatening complications (Mathews et al. 1999). However, there is only a little information about vascular anatomy of retroperitoneal teratomas. In one historical study abdominal arteriography has been performed on five cases of benign retroperitoneal teratomas. This investigation demonstrated a more avascular nature of these tumors (Brasch et al. 1978; Bruneton et al. 1980). Furthermore displacement of the “digestive arteries” was described. However, at surgery large and essential vessels may be partially or near completely surrounded by lobes, and fingers of the teratoma. As observed in both cases renal arteries and veins were stretched and depressed. In particular isolation of the large veins (superior mesenteric, portal, cava inferior, renal) requires careful isolation of the pseudocapsule, the use of loops and stepwise identification and dissection of many small and short veins originating from the teratoma and draining in the large retroperitoneal veins. Further on, vascular branches from the aorta and essential visceral and lumbar arteries have to be isolated and preserved (Brasch et al. 1978).

## Conclusion

Despite the description of retroperitoneal teratoma as an “unattached retroperitoneal tumor” anatomical relationships of high complexity have to been taken into account (Fig. 3) if surgery is performed.

## Figures and Tables

**Table 1:** Topography and involvement of important anatomical structures.

	Case 1	Case 2
Suspected origin	Right upper retroperitoneum	Left upper retroperitoneum
Maximum	Foramen epiploicum	Caudally to transverse colon
Pancreas	In front	Cranially, in front
Adrenal	Not identified	Cranially, in front
Transverse colon	Caudally to maximum	Cranially to maximum
Duodenum	Displaced to the right	Displaced to the right
Abdominal aorta	Behind, adherent	Behind, adherent
Inferior cava vein	Elongated, compressed, and displaced to the right	Behind, adherent
Superior mesenteric vein	Adherent	Not involved
Celiac trunk	Surrounded by tumor	Not involved
Superior mesenteric artery	Involved	Involved
Inferior mesenteric artery	Not involved	Involved
Renal vessels	Behind, adherent; left site elongated	Behind, adherent; left site elongated
